# Unusual *N*-Prenylation in Diazepinomicin Biosynthesis: The Farnesylation of a Benzodiazepine Substrate Is Catalyzed by a New Member of the ABBA Prenyltransferase Superfamily

**DOI:** 10.1371/journal.pone.0085707

**Published:** 2013-12-23

**Authors:** Tobias Bonitz, Florian Zubeil, Stephanie Grond, Lutz Heide

**Affiliations:** 1 Pharmaceutical Institute, Eberhard Karls-Universität Tübingen, Tübingen, Germany; 2 Institute of Organic Chemistry, Eberhard Karls-Universität Tübingen, Tübingen, Germany; University of Strathclyde, United Kingdom

## Abstract

The bacterium *Micromonospora* sp. RV115, isolated from a marine sponge, produces the unusual metabolite diazepinomicin, a prenylated benzodiazepine derivative. We have cloned the prenyltransferase gene *dzmP* from this organism, expressed it in *Escherichia coli*, and the resulting His_8_-tagged protein was purified and investigated biochemically. It was found to catalyze the farnesylation of the amide nitrogen of dibenzodiazepinone. DzmP belongs to the ABBA prenyltransferases and is the first member of this superfamily which utilizes farnesyl diphosphate as genuine substrate. All previously discovered members utilize either dimethylallyl diphosphate (C_5_) or geranyl diphosphate (C_10_). Another putative diazepinomicin biosynthetic gene cluster was identified in the genome of *Streptomyces griseoflavus* Tü4000, suggesting that the formation of diazepinomicin is not restricted to the genus *Micromonospora*. The gene cluster contains a gene *ssrg_00986* with 61.4% identity (amino acid level) to *dzmP*. The gene was expressed in *E. coli*, and the purified protein showed similar catalytic properties as DzmP. Both enzymes also accepted other phenolic or phenazine substrates. ABBA prenyltransferases are useful tools for chemoenzymatic synthesis, due to their nature as soluble, stable biocatalysts. The discovery of DzmP and Ssrg_00986 extends the isoprenoid substrate range of this superfamily. The observed prenylation of an amide nitrogen is an unusual biochemical reaction.

## Introduction

**Figure 1:**
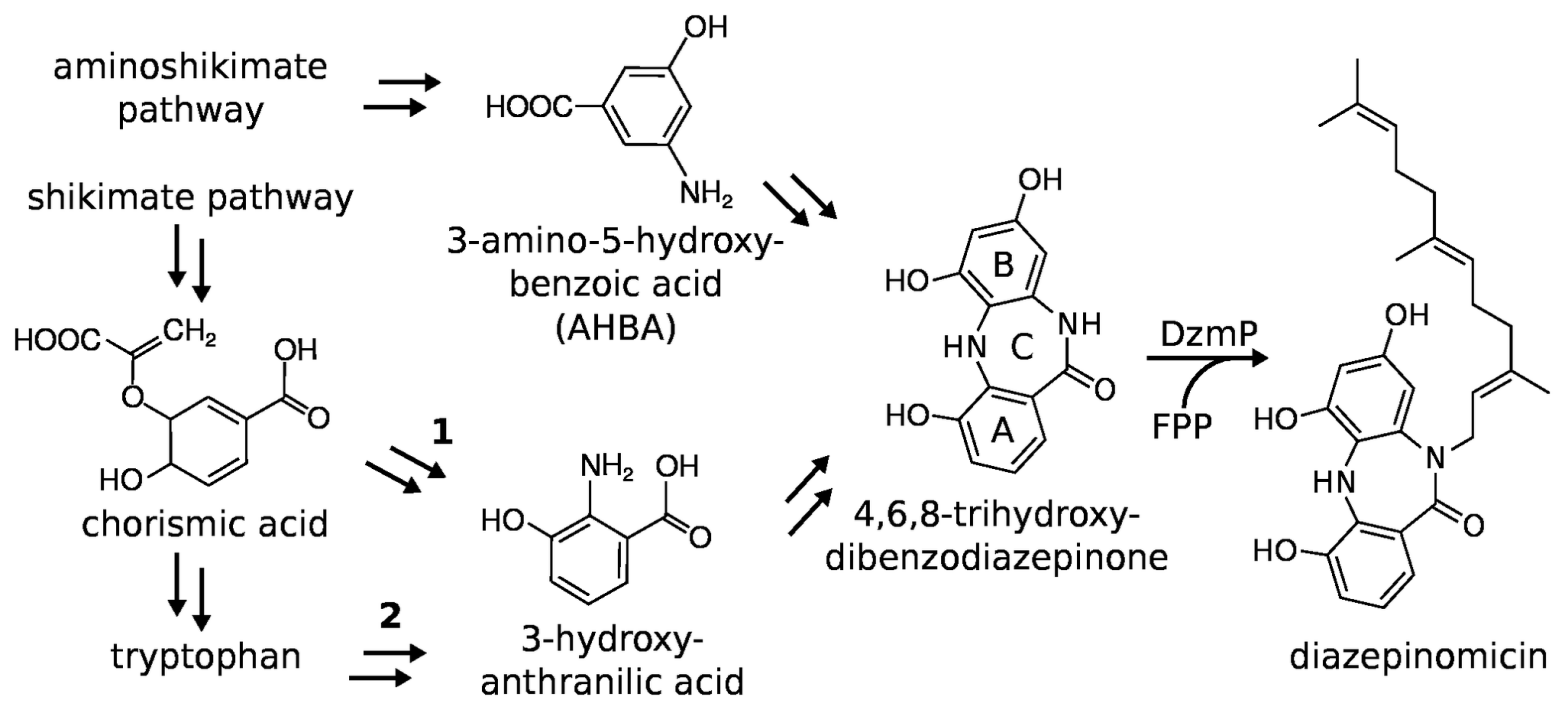
Diazepinomicin and its hypothetical biosynthetic pathway Hypothetical pathway encoded in the gene cluster of diazepinomicin, as proposed by McAlpine et al. [18]. 2) Degradation of tryptophan via the kynurenine pathway.

The isoprenylation of aromatic substrates is important in the biosynthesis of primary metabolites like ubiquinones, menaquinones and plastoquinones, but it also leads to a vast structural diversity of secondary metabolites in bacteria, fungi and plants [1]. Within the last ten years, a new superfamily of aromatic prenyltransferases, involved exclusively in bacterial and fungal secondary metabolism, has been discovered and extensively investigated [2-4]. Members of this superfamily are characterized by a new protein fold. It consists of a central barrel formed by ten antiparallel β-strands which contains the active center in its lumen and which is surrounded by a ring of solvent exposed α-helices [5]. Due to the αββα succession of secondary structure elements, this superfamily has been termed ABBA prenyltransferases [4]. It comprises two clearly distinct families [2], i.e. the fungal and bacterial indole prenyltransferases involved e.g. in ergot alkaloid biosynthesis [3,6], and the phenol / phenazine prenyltransferases predominantly involved in bacterial secondary metabolism [5,7,8]. Typically, ABBA prenyltransferases catalyze the *C*-prenylation of aromatic substrates, i.e. a reaction similar to a Friedel-Crafts alkylation, although a few members also catalyze *N*- or *O*-prenylations. The members of the ABBA superfamily are soluble, mostly Mg^2+^-independent catalysts, in contrast to the prenyltransferases of lipoquinone biosynthesis which are integral membrane proteins and require Mg^2+^ as cofactor. Their considerable promiscuity for different aromatic substrates makes them attractive tools for chemoenzymatic synthesis [3,4]. However, their genuine isoprenoid substrates have so far been strictly limited to either dimethylallyl diphosphate (DMAPP) or geranyl diphosphate (GPP), i.e. to substrates with five or ten carbon atoms. In order to expand the substrate range of this group of chemoenzymatic tools, we decided to search for ABBA prenyltransferases which might use farnesyl diphosphate (FPP) as genuine isoprenoid substrate. Our attention was drawn to the unusual natural product diazepinomicin (Figure 1). Its dibenzodiazepinone core is structurally unique in nature. The only related natural products containing benzodiazepine moieties are the pyrrolobenzodiazepines formed by bacteria, and the fungal metabolite cyclopenin [9].

Diazepinomicin has been independently isolated from different *Micromonospora* strains from different geographic locations of this world by Bristol-Myers Squibb [10,11], Thallion Pharmaceuticals (formerly Ecopia BioSciences) [12], Wyeth [13] and recently by Abdelmohsen et al. [14]. So far this compound has never been described outside of the genus *Micromonospora*. It was named BU-4664L, TLN-4601 (formerly ECO-4601) or diazepinomicin, and we will use the latter name hereafter.

Diazepinomicin binds to peripheral benzodiazepine receptors and has potent antitumor activity [15,16]. It has recently been investigated in a phase II clinical trial as anticancer agent [17]. 

Feeding studies have established that ring A of diazepinomicin (Figure 1) is derived from 3-hydroxy anthranilic acid [18]. This precursor can be formed by the kynurenine pathway which is used in actinobacteria for tryptophan catabolism [19,20]. Therefore, labeled tryptophan is incorporated into diazepinomicin [18,21,22]. However, the biosynthetic gene cluster for diazepinomicin (see below) contains genes for an even more efficient pathway which is suggested to lead from chorismate via 2-amino-2-deoxyisochorismate to 3-hydroxyanthranilate in three enzymatic steps [18]. The enzymes catalyzing this pathway are similar to PhzE and PhzD of phenazine biosynthesis [23] and to MxcC of myxochelin biosynthesis [24]. Furthermore McAlpine et al. [18] speculated that ring B of diazepinomicin is derived from 3-amino-5-hydroxybenzoic acid (AHBA), a metabolite derived from the aminoshikimate pathway [25].

The biosynthetic gene cluster for diazepinomicin has been cloned and sequenced from *Micromonospora* strain 046Eco-11 [18]. Based on a bioinformatic analysis of the DNA sequence, a detailed hypothesis of the biosynthetic pathway leading to diazepinomicin has been formulated [18], but the function of the individual genes and enzymes has not been investigated experimentally. *orf11* of this gene cluster shows sequence similarity to genes coding for ABBA prenyltransferases. It has therefore been speculated that the gene product of *orf11* may catalyze the farnesylation reaction in diazepinomicin biosynthesis [18].

**Figure 2:**
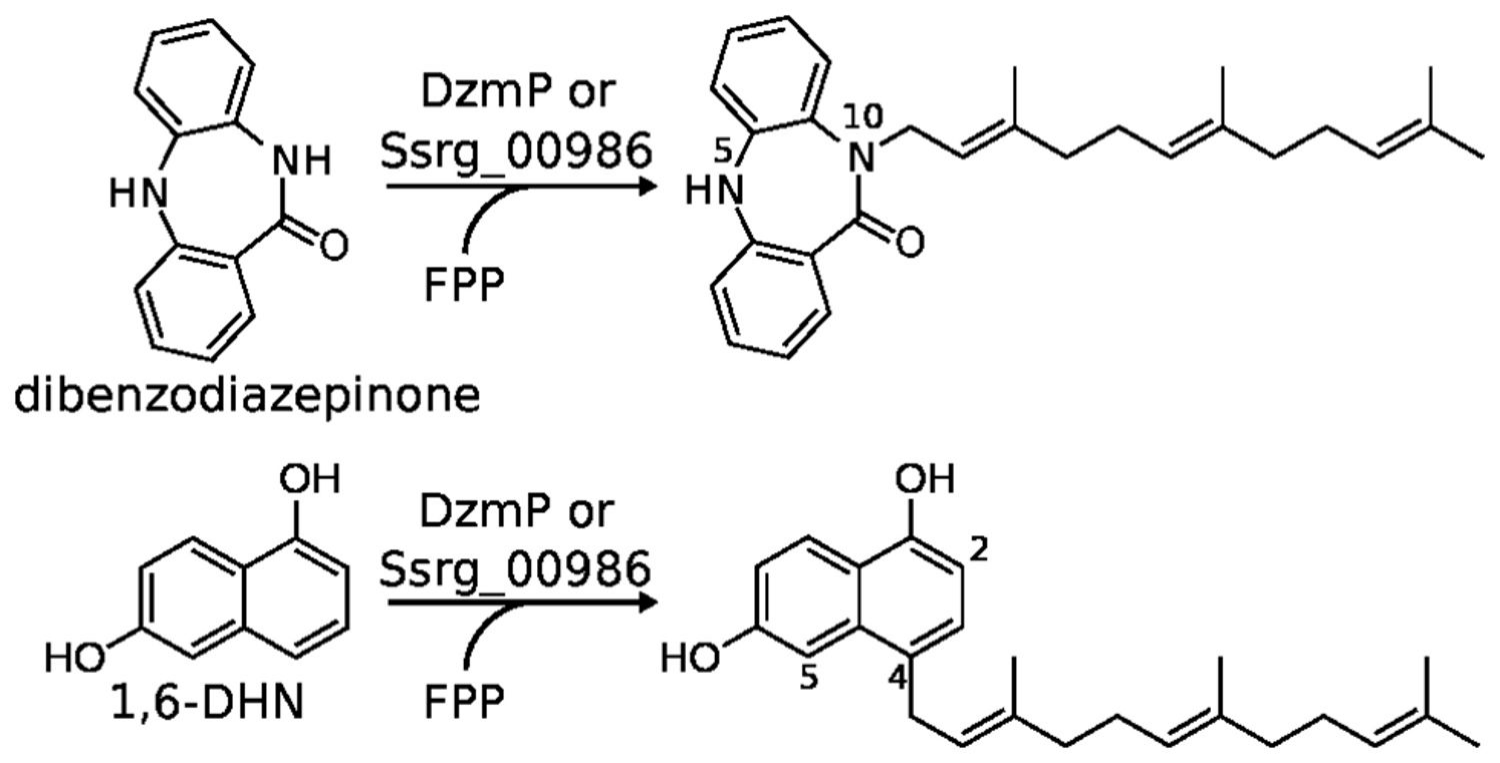
Reactions catalyzed by DzmP and Ssrg_00986. FPP, farnesyl diphosphate; 1,6-DHN, 1,6-dihydroxynaphthalene.

In the present study, we identified a close homolog of *orf11* (96.9% identity on the amino acid level) in the diazepinomicin producer strain *Micromonospora* sp. RV115 [14]. This gene (hereafter called *dzmP*) was expressed in *Escherichia coli* and the protein was purified. It was found to catalyze the farnesylation of the amide nitrogen of dibenzodiazepinone (Figure 2). In addition, homology searches by BLAST [26] revealed a gene cluster in *Streptomyces griseoflavus* Tü4000 with striking similarity to the diazepinomicin cluster. The *orf11* ortholog from this cluster was also expressed and purified, and was found to catalyze the same reaction as DzmP. Both enzymes were characterized and found to prenylate various phenolic and phenazine substrates, opening new possibilities for the chemoenzymatic synthesis of prenylated compounds. To our knowledge, DzmP is the first ABBA prenyltransferase which utilizes FPP as its genuine substrate. Both DzmP and its ortholog from *S. griseoflavus* Tü4000 catalyze the prenylation of an amide nitrogen, which is an unusual reaction in nature.

## Results

### Identification and cloning of two putative prenyltransferases from *Micromonospora* sp. RV115 and *Streptomyces griseoflavus* Tü4000

In the diazepinomicin gene cluster of *Micromonospora* strain 046Eco-11 identified by McAlpine et al. [18], the putative prenyltransferase gene *orf11* is flanked by the HMG-CoA synthase gene *orf10*, and by *orf12* coding for a putative sensor protein of a two-component regulatory system. Recently, diazepinomicin was isolated from the marine sponge-associated strain *Micromonospora* sp. RV115, in collaboration with one of the authors of the present study (SG) [14]. The structure of this compound was unequivocally confirmed by one- and two-dimensional NMR studies. We speculated that the gene clusters in both *Micromonospora* strains are very similar, and therefore we designed primers for the 3’ terminus of *orf10* and the 5’ terminus of *orf12*. Using genomic DNA of *Micromonospora* sp. RV115 as template, PCR readily gave a product of the expected size. Sequencing showed a coding sequence with very high similarity to *orf11* from *Micromonospora* strain 046Eco-11 (96.9% identity on the amino acid level). The *orf11* ortholog from *Micromonospora* sp. RV115 was termed *dzmP* (NCBI accession KC866371).

**Figure 3:**
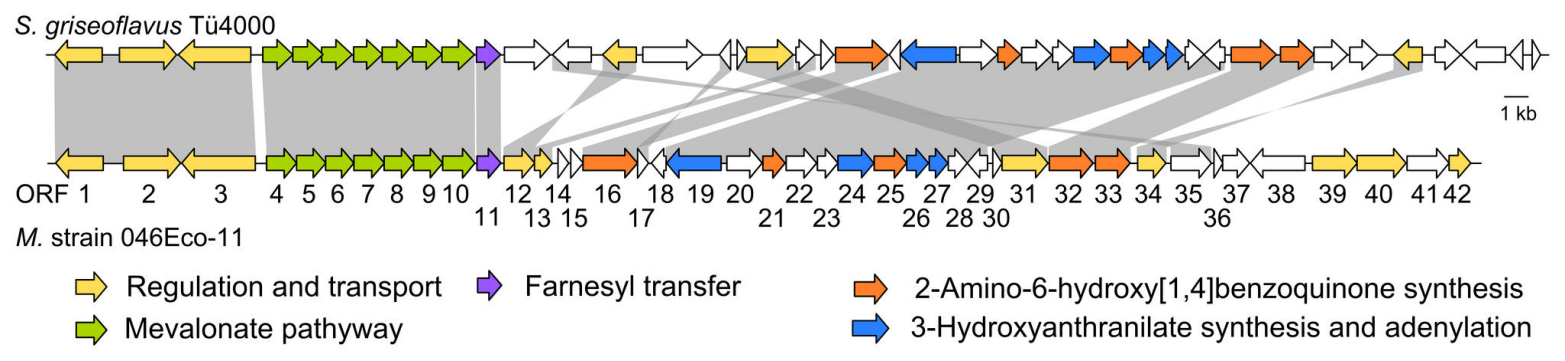
Comparison of the biosynthetic gene cluster of diazepinomicin in *Micromonospora* strain 046Eco-11 with the newly identified gene cluster in *Streptomyces griseoflavus* Tü4000. Homologous genes are connected by gray areas. The numbering and the suggested function of genes of *Micromonospora* strain 046Eco-11 follow the proposal by McAlpine *et al.* [18].

BLAST searches revealed in the genome of *Streptomyces griseoflavus* Tü4000 a gene termed *ssrg_00986* which is currently annotated as conserved hypothetical protein (NCBI accession ZP_07309813). This gene showed 61.4% identity (amino acid level) to *dzmP*. Inspection of the genes in the vicinity of *ssrg_00986* in the S. *griseoflavus* Tü4000 genome showed a gene cluster with high similarity to the diazepinomicin gene cluster from *Micromonospora* strain 046Eco-11 (Figure 3). This S. *griseoflavus* Tü4000 cluster contains orthologs of all genes for which an essential function in diazepinomicin biosynthesis has been suggested [18]. However, cultivation of *S. griseoflavus* Tü4000 in HI medium [18] and Bennett’s broth [14] followed by LC-MS analysis did not show any diazepinomicin production, suggesting that the gene cluster may be silent under the conditions employed. Nevertheless, we amplified the putative prenyltransferase gene *ssrg_00986* from genomic DNA of *S. griseoflavus* Tü4000. The correct DNA sequence was confirmed, and both this gene and *dzmP* were cloned into an expression vector for expression as N-terminally His-tagged proteins.

### Bioinformatic Sequence Analysis of DzmP and Ssrg_00986

**Figure 4:**
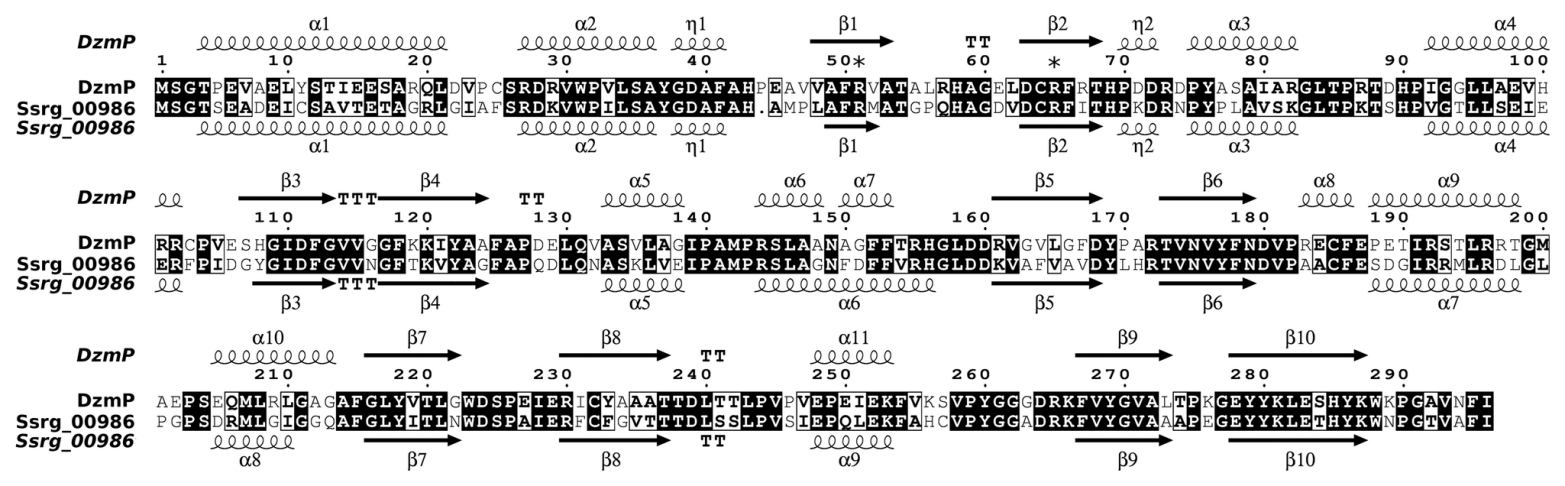
Amino acid alignment und secondary structure prediction for DzmP and Ssrg_00986 visualized by ESPript **[52]**. Secondary structure elements are: α, α-helices; η, 3_10_-helices; β, β-strands; TT, strict β-turns. Strict sequence identity is shown by a black box with white characters, and similarity is shown by bold characters in a black frame. The position of the two arginine residues typical for Mg^2+^-independent ABBA prenyltransferases are indicated by asterisks.

**Figure 5:**
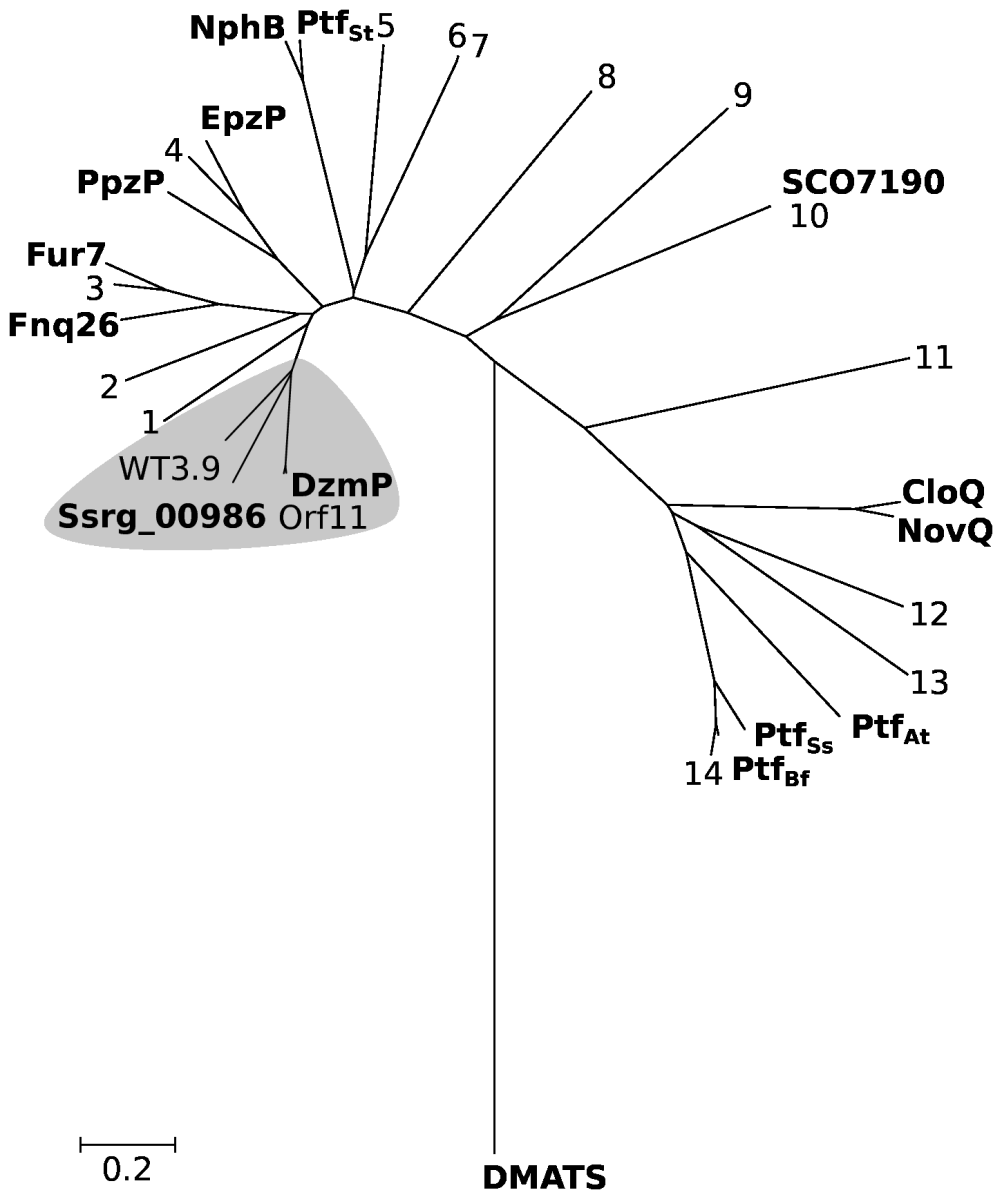
Evolutionary tree of aromatic prenyltransferases of the CloQ / NphB family (= phenol / phenazine prenyltransferases [2]). The branch highlighted in gray represents the new prenyltransferases investigated in this study. The evolutionary tree was generated with MEGA4 using the default parameters for pairwise (multiple) alignment. Phylogenetic reconstruction was carried out using the neighbor-joining method. Names of biochemically investigated enzymes [2] are shown in bold, and further uncharacterized NCBI database entries are: 1, ZP_10450727; 2, ABS50461 and ABS50489; 3,CCK32327; 4, AEW22941; 5, CAL34106; 6, ABS50462; 7, ABS50490; 8, YP_005045476; 9, YP_006808349; 10, ZP_06526769; 11, ZP_09171437; 12, XP_002847323; 13, XP_002143864 and 14, CCD48995.

The Genes *dzmP* and *ssrg_00986* code for proteins of 296 and 295 amino acids, respectively. Secondary structure prediction shows for both gene products the five-fold ααββ repeat which is typical for the ABBA prenyltransferases (Figure 4). A phylogenetic analysis (Figure 5) places DzmP, Orf11 and Ssrg_00986 into a new, separate branch of the previously described family of phenol / phenazine prenyltransferases [2]. DzmP and its orthologs are quite similar to the hydroxynaphthalene prenyltransferases NphB [27] and Fnq26 [28], and to the phenazine prenyltransferases PpzP [29] and EpzP [8,30] (average sequence identity ~ 43%). While this study was in progress, a further gene cluster with close similarity to the diazepinomicin cluster was deposited in GenBank by the group of Zhongjun Qin from the Shanghai Institutes for Biological Sciences. This cluster (NCBI accession JQ432566) was obtained from a strain termed *Streptomyces* sp. WT3. It contains a gene named *wt3.9* which codes for a 296 aa protein with close similarity to DzmP and Ssrg_00986 (Figure 5). No further close orthologs of DzmP, and no further gene clusters with close similarity to the diazepinomicin cluster, are currently deposited in GenBank.

DzmP and Ssrg_00986 (Figure 5), as well as Orf11 and Wt3.9, contain arginine residues in position 51 and 64. X-ray crystallographic studies [7] as well as modeling studies [4] have suggested that these residues are essential for the cofactor-independent binding of the α-phosphate of the isoprenoid substrate, and therefore are characteristic for ABBA prenyltransferases which are independent of Mg^2+^. In contrast, Mg^2+^-dependent ABBA prenyltransferases such as NphB [5] and Ptf_St_ [31] contain serine residues in these positions. DzmP and Ssrg_0986 are therefore expected to catalyze a prenyl transfer independent of the presence of magnesium ions.

### Expression and purification of DzmP and Ssrg_00986

**Figure 6:**
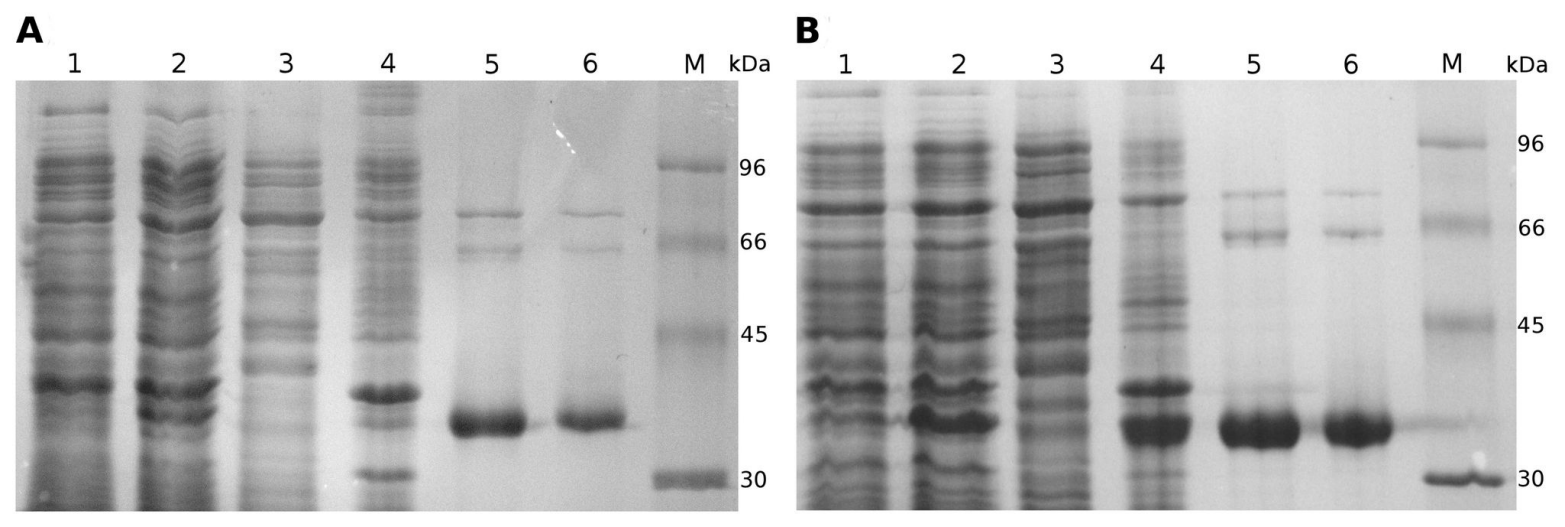
Purification of DzmP (A) and Ssrg_00986 (B) after expression as His_8_-tagged fusion proteins. *Lane 1*, total protein before IPTG induction; lane *2*, total protein after IPTG induction; lane *3*, soluble protein after IPTG induction; lane *4*, insoluble protein after IPTG induction; lane *5*, protein after Ni^2+^ affinity chromatography; lane *6*, protein after buffer exchange over Sephadex G-25; lane *M*, molecular mass standards. The calculated masses are 34.8 kDa for both enzymes. The 12% polyacrylamide gel was stained with Coomassie Brilliant Blue R-250.

DzmP and Ssrg_00986 were expressed as His-tagged proteins in *E. coli*, readily yielding soluble proteins which were purified by Ni^2+^ affinity chromatography (Figure 6). From 1 L of culture 9.5 mg DzmP and 7.5 mg Ssrg_00986 were obtained.

### Prenyltransferase activity of DzmP and Ssrg_00986

In the hypothetical biosynthetic pathway of diazepinomicin suggested by McAlpine et al. [18], the farnesylation of 4,6,8-trihydroxy-dibenzodiazepinone (Figure 1) is suggested as the final step. Commercially, only non-hydroxylated dibenzodiazepinone is available, and we therefore tested this compound (Figure 2) as substrate. Furthermore, 1,6-dihydroxynaphthalene (1,6-DHN) was tested, since this compound is known to be accepted by NphB [27], a prenyltransferase with similarity to DzmP (Figure 5). Using farnesyl diphosphate (FPP) as isoprenoid substrate, an enzymatic prenylation of both aromatic compounds by DzmP and, with lower activity, by Ssrg_00986, was readily observed by HPLC-UV and HPLC-MS. Dimethylallyl diphosphate (DMAPP) and geranyl diphosphate (GPP) were converted with much lower reaction velocity (6% and 12% compared to FPP, respectively), and therefore the investigations were continued with FPP. Enzymatic product formation showed linear dependence on time for at least 30 min, and linear dependence on protein amount at least up to 12 µM. The addition of 100 mM NaCl increased activity by 10%, whereas the addition of glycerol had no effect. Product formation was readily detectable in the absence of magnesium ions, but the addition of 2 mM Mg^2+^ increased the activity of DzmP approximately 1.5 fold and the activity of Ssrg_00986 approximately 3 fold compared to assays without Mg^2+^. Addition of EDTA (1 mM) did not influence the activity. Maximal product formation for DzmP was observed at pH 8.0, with half-maximal values at pH 5.8 and 10.3. Ssrg_00986 showed maximal activity at pH 7.5, with half-maximal values at pH 6.3 and 8.2.

### Structural identification of the farnesylation product of dibenzodiazepinone

Prenylation of dibenzodiazepinone by an ABBA prenyltransferase may occur at many different positions of the molecule. Each of the two aromatic rings (Figure 2) offers four unsubstituted carbons for *C*-prenylation, in a reaction which would resemble the prenylation of the indole nucleus of L-tryptophan by different indole prenyltransferases [32,33] or the *C*-prenylation of a dihydrophenazine derivative by PpzP and EpzP [8,29]. Notably, PpzP and EpzP show close similarity to DzmP (Figure 5). However, a few ABBA prenyltransferases catalyze *N*-prenylations [34-37]. In the dibenzodiazepinone molecule the nitrogen atom which is chemically most reactive for alkylation reactions is N-5 (Figure 2). In contrast the amide nitrogen (N-10) is expected to be much less reactive, and enzymatic prenylations of an amide nitrogen are very unusual.

Prenylation of dibenzodiazepinone with FPP under catalysis of DzmP yielded a single product which showed the mass of a mono-farnesylated product. In order to determine the substitution position, the assay was scaled up to 20 mL and the enzymatic product was purified on preparative scale. By APCI-HRMS, the molecular formula of the reaction product was deduced to be C_28_H_34_N_2_O ([M+H^+^]: C_28_H_35_N_2_O^+^, *m/z* calc. 415.27439, found 415.27433, Δ 0.15 ppm). MS-MS showed a fragment C_14_H_11_N_2_O^+^ ([M^+^], *m/z* calc. 223.08659, found 223.08678, Δ 0.87 ppm) indicating the C-1’-C-2’ cleavage of the farnesyl chain. A proposed mechanism of the fragmentation is given in the Supplementary (Figure S1). Full ^1^H and ^13^C NMR spectroscopic data of the product as well as selected ^1^H-^1^H COSY, NOESY and ^1^H HMBC correlations are given in the Supplementary (Figure S2 and Table S1). The position of the farnesylation was unequivocally confirmed by NOESY (τ_m_ = 1 s). NOESY signals of H-1’ (δ = 4.48 ppm) and H-9 (δ = 7.26 ppm), H-5 (δ = 7.82 ppm) and H-6 (δ = 7.10 ppm), H-5 and H-4 (δ = 7.04 ppm) as well as a HMBC signal of H-1’ and the carbonyl carbon C-11 (δ = 167.5 ppm) prove the farnesylation of dibenzodiazepinone at the amide nitrogen in position 10 (Figure 2).

The farnesylated products generated from dibenzodiazepinone under catalysis of DzmP and Ssrg_00986 showed exactly the same retention time under different HPLC conditions, as well as the same mass and fragmentation in HPLC-MS-MS analysis. This indicates that both enzymes catalyze the same reaction.

### Structural identification of the major farnesylation product of 1,6-dihydroxynaphthalene

**Figure 7:**
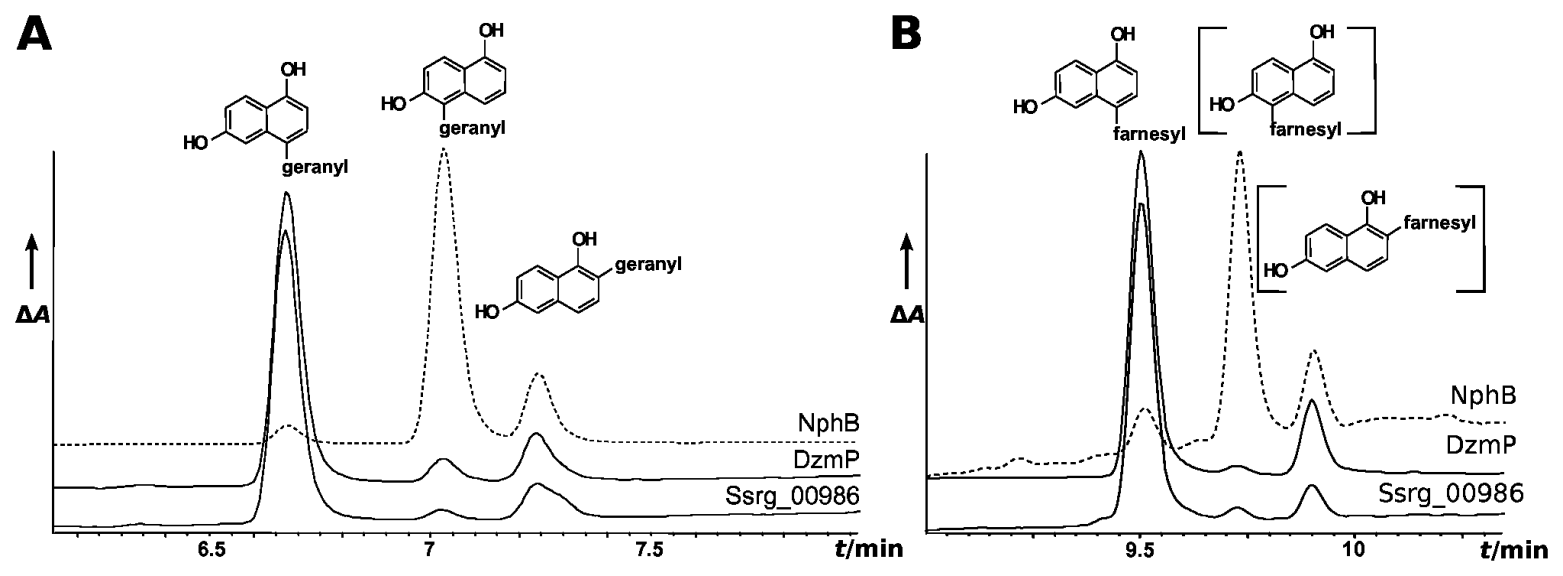
Geranylation (A) or farnesylation (B) of 1,6-DHN under catalysis of NphB, DzmP and Ssrg_00986. Reaction products were analyzed by HPLC with UV detection at 286 nm. Absorbance was scaled differently for each enzyme in order to improve visibility of the product peaks.

The reaction of 1,6-DHN with FPP under catalysis of DzmP yielded one major product (Figure 7B; R_t_ = 9.5 min) and two minor products (R_t_ = 9.7 min and 9.9 min), each showing the mass of a mono-farnesylated 1,6-DHN. A preparative scale assay was carried out and the major product was isolated for structural elucidation. The molecular formula of the reaction product was deduced to be C_25_H_32_O_2_ ([M+H^+^]: C_25_H_33_O_2_^+^, calc. 365.24751, found 365.24741, Δ 0.27 ppm) by HPLC-ESI-HRMS. The fragment C_11_H_9_O_2_^+^ ([M^+^], calc. 173.05971, found 173.05969, Δ 0.1 ppm) indicated the C-1’-C-2’ cleavage of the farnesyl chain. A proposed mechanism of the fragmentation is given in the Supplementary (Figure S3). The position of the farnesylation of 1,6-DHN was determined using unidimensional (^1^H NMR, ^13^C NMR) and multidimensional (^1^H-^1^H COSY, ^1^H, ^13^C HSQC and ^1^H, ^13^C HMBC) NMR spectroscopy (Figure S4 and Table S2), revealing coupling patterns of the aromatic protons in ^1^H NMR and unambiguous COSY signals. The coupling pattern indicates H-5 (δ = 7.20 ppm; d, 2.4 Hz) to be in *meta* position of H-7 (δ = 7.03 ppm; dd, 2.4 and 9.0 Hz) and H-8 (δ = 8.05 ppm; d, 9.0 Hz) to be in *ortho* position to H-7 as well as H-2 (δ = 6.58 ppm; d, 7.6 Hz) to be in *ortho* position of H-3 (δ = 7.07 ppm; d, 7.6 Hz). Because of the characteristical chemical shifts of H-2 and H-8, and due to the close similarity to the published NMR data of 4-geranyl-1,6-DHN [27], the position of the farnesylation could be deduced to be at C-4 (Figure 2). The products formed by Ssrg_00986 from the same substrates showed the same retention time as the DzmP products in different HPLC systems, and showed the same mass and fragmentation in HPLC-MS-MS analysis. This suggested that the products from both enzymes are identical.

### Comparison of the prenylation reactions of 1,6-DHN catalyzed by NphB, DzmP and Ssrg_00986

NphB catalyzes the geranylation of a (so far unknown) hydroxynaphthalene substrate in the biosynthesis of naphterpin [27]. Using the artificial substrate 1,6-DHN, it preferentially prenylates the 5-position. In clear contrast, we found that DzmP preferentially farnesylates the 4-position of 1,6-DHN (see above). This difference prompted us to express and purify NphB following the same procedure as for DzmP and Ssrg_00986, and to compare the reaction products of NphB, DzmP and Ssrg_00986 upon incubation with 1,6-DHN and either GPP or FPP.

As expected, NphB preferentially accepted GPP as substrate; FPP was converted 30 times more slowly. In contrast, DzmP preferentially accepted FPP, and GPP was converted 8 times more slowly. As published previously, NphB forms three geranylated products from 1,6-DHN (Figure 7A). The major product has been identified as 5-geranyl-1,6-DHN, with 2-geranyl-1,6-DHN as the second most abundant and 4-geranyl-1,6-DHN as the least abundant product [27]. As shown in Figure 7A, also DzmP forms three products from GPP and 1,6-DHN, and these showed the same HPLC retention time, and the same mass and fragmentation pattern, as the products formed by NphB. In case of DzmP the peak corresponding to 4-geranyl-1,6-DHN is the dominant product, and 5-geranyl-1,6-DHN is the least abundant product. A similar formation of three products is observed when FPP is used as substrate (Figure 7B). 4-farnesyl-1,6-DHN is the major product formed by DzmP, while this compound is the least abundant product formed by NphB. Therefore, NphB and DzmP show a different regioselectivity in the prenylation of 1,6-DHN. The product spectrum of Ssrg_00986, both with GPP and FPP, was found to be very similar to that of DzmP (Figure 7A and B), confirming the close similarity of the reaction catalyzed by both enzymes.

### Kinetic investigations of the prenylation of 1,6-DHN and dibenzodiazepinone by DzmP and Ssrg_00986

The reactions of DzmP and Ssrg_00986 with dibenzodiazepinone and 1,6-DHN as aromatic substrates, and with GPP and FPP as isoprenoid substrates, were investigated. Kinetic constants were calculated using nonlinear regression. Depending on the substrates used, a slightly better fit was obtained by using the equations for sigmoidal curve (indicating cooperativity) or for a substrate inhibition kinetic [38], rather than by using the Michaelis-Menten equation (Figure S5). Using 1,6-DHN, calculations were based on the formation of the 4-prenylated product (i.e. the major product). Using 1 mM FPP and different concentrations of 1,6-DHN, both DzmP and Ssrg_0986 gave sigmoidal curves. K_0.5_ values for 1,6-DHN were calculated as 18 ± 1 and 96 ± 11 µM, respectively. For DzmP, k_cat_ was determined as 4.5 ± 0.1 s^-1^ × 10^-3^, comparable to the value of 4.2 ± 0.2 s^-1^ × 10^-3^ published for the reaction of GPP and 1,6-DHN catalyzed by NphB [27]. For Ssrg_00986, k_cat_ was 0.4 ± 0.01 s^-1^ × 10^-3^.

Using 1 mM FPP and different concentrations of dibenzodiazepinone, substrate inhibition (K_i_ = 655 ± 213 µM) was observed. The K_m_ and k_cat_ values of DzmP for dibenzodiazepinone were determined as 133 ± 30 µM and 0.68 ± 0.09 s^-1^ × 10^-3^, respectively. For Ssrg_00986, reaction velocity was at least 10 times lower, preventing the reliable measurement of kinetic constants.

Using 1 mM 1,6-DHN and different concentrations of FPP, the plot of reaction velocity over substrate concentration could not be adequately fitted to a Michaelis-Menten, sigmoidal or substrate inhibition kinetic for DzmP. Half-maximal reaction velocity was observed at approximately 20 µM FPP. With GPP as isoprenoid substrate, a sigmoidal curve was obtained, and K_m_ and k_cat_ were calculated as 4.9 ± 0.9 µM and 0.5 ± 0.01 s^-1^ × 10^-3^, respectively.

For Ssrg_00986, a precise comparison of the kinetic constants for FPP and GPP was hampered by the low reaction velocity. However, the enzyme did not show a clear preference for FPP: for both isoprenoid substrates, half-maximal reaction velocities were observed at approximately 45 µM, and maximal turn-over rates were approximately 0.5 s^-1^ × 10^-3^.

### Prenylation of further aromatic substrates

 In order to investigate the promiscuity of DzmP for different aromatic substrates, the enzyme was incubated with FPP and either 2,7-dihydroxynaphthalene (2,7-DHN), 5,10-dihydrophenazine-1-carboxylic acid (dihydro-PCA), flaviolin (2,5,7-trihydroxy-1,4-naphthoquinone) and the isoflavonoid genistein. These compounds have been described to be accepted by ABBA prenyltransferases with sequence similarity to DzmP, e.g. by NphB [27], PpzP [29] or Fnq26 [28]. As determined by HPLC-UV and HPLC-MS-MS, all of these four compounds were converted to mono-farnesylated products, with turn-over rates of approximately 0.57, 0.34, 0.21 and 0.11 s^-1^ × 10^-3^, respectively. 2,7-DHN and flaviolin yielded a single product, genistein yielded two, and in the prenylation of dihydro-PCA the major product was accompanied by four side products of the same mass, suggesting that farnesylation may occur at different positions of the molecule (Figure S6).

## Discussion

ABBA prenyltransferases are useful tools for chemoenzymatic synthesis, due to their nature as soluble, stable biocatalysts which is in contrast to the membrane-bound nature of prenyltransferases of lipoquinone biosynthesis. Most ABBA prenyltransferases are independent from magnesium as cofactor which is desirable since magnesium ions accelerate the non-enzymatic hydrolysis of prenyl diphosphates [39]. 

The active center of the ABBA prenyltransferases is located within the central barrel which is formed by ten antiparallel β-strands. Nearly all of the amino acids interacting with the substrates are part of these β-strands, creating a spatially restricted environment [5-7]. After the discovery of CloQ and NphB which utilize DMAPP (C_5_) or GPP (C_10_) as isoprenoid substrates, respectively [5,7,40], speculations have been offered which structural features of an ABBA prenyltransferase determine the chain length specificity for the isoprenoid substrate [4,41]. E.g. it has been suggested that R66 and E281 in CloQ form salt bridges which sterically hinder the accommodation of geranyl diphosphate and thereby restrict the isoprenoid substrate to five carbons, i.e. to dimethylallyl diphosphate [4]. Notably, however, the same residues are now found in DzmP in form of R65 and E283 (Figure S7), as suggested by modeling of both CloQ and DzmP using the Phyre 2 [42] and the ASC server [43]. This contradicts the above hypothesis that these residues restrict the chain length of the isoprenoid substrate. Further X-ray crystallographic investigations and mutagenesis experiments will be required in order to elucidate how the different chain length specificities of ABBA prenyltransferases are determined.

The prenylation of an amide nitrogen has not previously been described from an enzyme of the ABBA prenyltransferase superfamily. Prenylated amide nitrogens are very rarely found in nature. Among the few exceptions are *N*-prenylated xanthine derivatives which have been described from plants [44], as well as some protozoan secondary metabolites [45]. 

The keto group of the dibenzodiazepinone is subject to a keto-enol tautomerism. Recent X-ray crystallographic investigations suggest that the keto (= amino-oxo) tautomer is the dominant form, compared to the enol (= hydroxyimine) form [46]. Only the keto form offers the possibility to abstract a proton from the amide nitrogen, which is likely to be required before the alkylation of this heteroatom can take place.

The regiospecific farnesylation of N-10 of dibenzodiazepinone by DzmP supports the hypothesis by McAlpine et al. [18] that this *N*-prenylation reaction may be the last step of diazepinomicin biosynthesis (Figure 1). The suggested genuine substrate, i.e. 4,6,8-trihydroxy-dibenzodiazepinone, is likely to be more reactive towards *N*-alkylation due to the OH-groups which may also contribute to the binding of the substrate in the active center. The absence of these groups in the artificial substrate dibenzodiazepinone (Figure 2) may explain the relatively low catalytic turnover and high K_m_ value observed for that compound.

The dibenzodiazepinone structure has been described as unique in nature [18], and has so far only been found in the genus *Micromonospora*. The discovery of gene clusters with similarity to the diazepinomicin cluster in *Streptomyces griseoflavus* Tü4000 (Figure 3), and in *Streptomyces* sp. WT3 (NCBI accession JQ432566) may indicate that similar compounds can be found also in *Streptomyces* strains. However, the exact structure of such compounds, including the length of their isoprenoid chain, remains to be elucidated.

## Conclusion

DzmP is the first member of the ABBA prenyltransferase superfamily which utilizes farnesyl diphosphate (FPP; C_15_) as preferred substrate. All previously discovered members utilize either DMAPP (C_5_) or GPP (C_10_) as their preferred substrate. The discovery of DzmP now provides a welcome extension of the isoprenoid substrate range of this superfamily. Furthermore, benzodiazepines like dibenzodiazepinone form a new class of prenyl acceptor substrates of ABBA prenyltransferases. The discovery of DzmP shows that the substrate range of the previously described phenol / phenazine prenyltransferase family [2] extends beyond these compound classes, and may be further expanded when other currently uncharacterized GenBank entries with similarity to this family (Figure 5) are investigated.

## Experimental Section

### Strains and culture conditions

*Micromonospora* sp. RV 115 [14] was provided by U. Hentschel, Würzburg, Germany. *Streptomyces griseoflavus* Tü4000 [47] was provided by W. Wohlleben, Tübingen, Germany. They were grown in liquid YMG medium or on solid MS medium. For production of secondary metabolites, the media described by McAlpine et al. [18] and Abdelmohsen et al. [14] were used. *Escherichia coli* XL1 Blue MRF (Stratagene, Heidelberg, Germany) was used for cloning and was grown in liquid or on solid (1.5% agar) Luria-Bertani medium at 37°C. Kanamycin (50 µg mL^-1^) was used for selection of recombinant strains.

#### Chemicals

Geranyl diphosphate and farnesyl diphosphate were synthesized as described by Woodside et al. [48]. Phenazine-1-carboxylic acid (PCA) was purchased from InFarmatic (Budapest, Hungary) and dihydrophenazine-1-carboxylic acid was generated as described by Saleh et al. [29]. Genistein was purchased from TCI Europe (Eschborn, Germany). 1,6-Dihydroxynaphthalene (1,6-DHN) and 5,10-dihydro-11*H*-dibenzo[*b*,*e*][1,4]diazepin-11-one were bought from Sigma Aldrich, Steinheim, Germany. 2,7-Dihydroxynaphthalene (2,7-DHN) was purchased from Acros Organics (Geel, Belgium).

#### Genetic procedures

Standard methods for DNA isolation and manipulation were performed as described by Kieser et al. [49] and Sambrook et al. [50]. DNA fragments were isolated from agarose gels by using a PCR purification kit (Qiagen; Hilden, Germany). Genomic DNA was isolated from *Micromonospora* and *Streptomyces* strains by lysozyme treatment and phenol / chloroform extraction as described by Kieser et al. [49]. Restriction enzymes were purchased from New England BioLabs, Ipswich, MA.

#### Sequence analysis

Database searches were performed with BLAST (http://www.ncbi.nlm.nih.gov/). Secondary structure predictions were performed with Phyre 2 [42], and sequences were aligned with ClustalX [51] and visualized with ESPript [52] or Jalview [53]. Phylogenetic trees were built with MEGA (http://www.megasoftware.net/). Protein structures were modeled with the Schrödinger suite (http://www.schrodinger.com/), examined with Pymol (http://www.pymol.org/) or the ASC server (http://asc.informatik.uni-tuebingen.de/).

### Overexpression and purification of DzmP, Ssrg_00986 and NphB

*dzmP* and its flanking sequences were amplified from genomic DNA of *Micromonospora* sp. RV115 using the primers orf10_F (5’-cgc gtc tac gag ccg cgM tag-3’) and orf12_R (5’-GAH SAK CAG YRC CGC BGC CAC-3’). The PCR product was used for sequencing (NCBI accession KC866371). For the construction of expression plasmids, *dzmP* and *ssrg_00986* were amplified using genomic DNA of *Micromonospora* sp. RV115 and *S. griseoflavus* Tü4000 as templates. The following primers were used for *dzmP*: dzmP_F: (5’-cat gcc atg gca tgt ccg gaa ctc ccg agg-3’) and dzmP_R: (5’-cgg aat tca aat gaa gtt cac cgc gcc c-3’). The underlined letters represent NcoI and EcoRI restriction sites, respectively. The resulting PCR fragment was cloned into the pGEM-T easy vector (Promega; Mannheim, Germany). After restriction analysis and sequencing the plasmid was digested with NcoI and EcoRI and the insert ligated into plasmid pHis_8_ [54] digested with the same restriction enzymes. Primers for amplification of *ssrg_00986* were ssrg_00986_pHis_8__F (5’-CTG CGC GAA TAC CGG AGC TCT GAT CAG ATG AGG CCA CAG T-3’) and ssrg_00986_pHis_8__R (5’-GCC TCA ATC GCT TGG GAT CCT ACA TGT CCG GAA CCT CCG AG-3’). The underlined letters represent BamHI and SacI restriction sites, respectively. The resulting PCR fragment was digested with BamHI and SacI and ligated into plasmid pHis_8_ digested with the same restriction enzymes.

The amino acid sequence of NphB was translated into the nucleotide sequence optimized for expression in *E. coli* with the Gene Designer Tool, provided with a His_8_ tag similar to pHis_8_ [54] and synthesized commercially by DNA2.0 (Basel, Switzerland). The resulting *dzmP*, *ssrg_00986* and *nphB* expression plasmids were verified by restriction mapping and sequencing.

*E. coli* Rosetta (DE3) pLysS cells harboring the respective expression plasmid were cultivated in 2 liters of liquid TB medium containing kanamycin (50 µg mL^-1^) and chloramphenicol (25 µg mL^-1^) and grown at 37°C to an A_600_ of 0.6. The temperature was lowered to 20°C, and IPTG was added to a final concentration of 0.5 mM. The cells were cultured for a further 15 h at 20°C. Cells were harvested by centrifugation for 20 min at 2,700 × g at 4°C and the pellet (40 g) was resuspended in 100 mL of lysis buffer (50 mM Tris-HCl, pH 8.0, 1 M NaCl, 10% glycerol, 10 mM β-mercaptoethanol, 20 mM imidazole, 0.5 mg mL^-1^ lysozyme, 0.5 mM phenylmethylsulfonyl fluoride). After stirring at 4°C for 30 min, cells were ruptured with a Branson sonifier at 4°C. Debris and membranes were removed by centrifugation at 38,720 × g for 45 min. The supernatant was applied to a nickel-nitrilotriacetic acid-agarose resin column (GE Healthcare) according to the manufacturer’s instructions, using a linear gradient of 20 to 250 mM imidazole (in 50 mM Tris-HCl, pH 8.0, 500 mM NaCl, 10% glycerol, 10 mM β-mercaptoethanol) in 60 min for elution. Subsequently, a buffer exchange was carried out by PD10 columns (Amersham Biosciences), which were eluted with 50 mM Tris-HCl, pH 8.0, 1 M NaCl, 10% glycerol and 2 mM 1,4-dithiothreitol. Approximately 19 mg of purified DzmP, 15 mg Ssrg_00986 and 19 mg NphB were obtained from 2 liters of cultures.

### Assay for prenyltransferase activity

The reaction mixture (100 µL) contained Tris-HCl pH 7.5 (100 mM), aromatic substrate (0.5 mM), isoprenoid substrate (0.4 mM), MgCl_2_ (2 mM) and either DzmP (0.3 µM) or Ssrg_00986 (0.6 µM). After incubation of the assay for 15 min at 30°C (for DzmP) or 30 min (for Ssrg_00986), the reaction mixture was cooled to 0°C. Ethyl acetate (200 µL) was added, and after vortexing and centrifugation 175 µL of the organic layer was transferred to an Eppendorf tube. The solvent was evaporated, and the residue was dissolved in methanol (50 µL) and analyzed by HPLC (Agilent 1100 series; Waldbronn, Germany) using a Kinetex column (2.6 µm, 100 × 4.6 mm; Phenomenex, Aschaffenburg, Germany) at a flow rate of 1 mL min^-1^ with a linear gradient from 40 to 100% of solvent B in 20 min (solvent A: water/formic acid (999:1); solvent B: methanol/formic acid (999:1)) and detection at 230 nm for dibenzodiazepinone, 272 nm for genistein, 286 nm for 1,6-DHN and 2,7-DHN, 307 nm for flaviolin and 365 nm for PCA. Additionally, a UV spectrum from 200 to 400 nm was logged by a photodiode array detector. The absorbance at 230 and 286 nm were used for quantitative analysis of dibenzodiazepinone and 1,6-DHN. For determination of the K_m_ values for 1,6-DHN and dibenzodiazepinone, FPP was kept at a constant concentration of 1 mM. For determination of the K_m_ value for GPP and FPP, 1,6-DHN was kept at 1 mM.

### Analysis by HPLC-MS

 The extracts were examined with HPLC-MS and HPLC-MS-MS analysis using a Nucleosil 100-C18 column (3 µm, 100 × 2 mm) coupled to an ESI mass spectrometer (LC/MSD Ultra Trap System XCT 6330; Agilent Technology). Analysis was carried out at a flow rate of 0.4 mL min^-1^ with a linear gradient from 10 to 100% of solvent B in 15 min (solvent A: water/formic acid (999:1); solvent B: acetonitrile/formic acid (999.4:0.6)). Detection was carried out at 230, 260, 280, 360, and 435 nm. Electrospray ionization (positive and negative ionization) in Ultra Scan mode with capillary voltage of 3.5 kV and heated capillary temperature of 350°C was used for analysis.

### Preparative enzymatic synthesis for structure elucidation

 50 mL (1,6-DHN) or 20 mL (dibenzodiazepinone) of the reaction mixture for prenyltransferase activity with addition of sodium ascorbate (10 mM) was incubated at 20°C overnight and extracted twice with dichloromethane (5 mL). The solvent was evaporated and the products were purified by preparative HPLC using a Multospher 120 RP 18 column (5 µm, 250 × 8 mm, Ziemer Chromatographie, Langerwehe, Germany) at a flow rate of 2.5 mL min^-1^. An isocratic elution with 75% (products of 1,6-DHN) or 80% (product of dibenzodiazepinone) of solvent B was used (solvent A: water; solvent B: methanol).

### Structural elucidation of the products of the DzmP reactions

The ^1^H and ^13^C nuclear magnetic resonance (NMR) spectra were recorded on a Bruker AMX-600 spectrometer operating at a proton frequency of 600.1 MHz. All spectra were recorded at room temperature. Chemical shifts are expressed in parts per million (ppm, δ) and referenced to tetramethylsilane (TMS, δ = 0 ppm). Coupling constants are expressed in Hz. The correct assignment of the chemical shifts was confirmed by application of two-dimensional correlation measurements, including correlation spectroscopy (COSY), heteronuclear single quantum coherence (HSQC), heteronuclear multiple bond coherence (HMBC) and if required by nuclear Overhauser enhancement spectroscopy (NOESY). High resolution mass spectra were measured using either electron spray ionization or atmospheric-pressure chemical ionization on a Bruker Maxis 4G.

The systematic names for the enzymatic products are 5,10-dihydro-10-[(2*E*,6*E*)-3,7,11-trimethyl-2,6,10-dodecatrienyl]-11*H*-dibenzo[*b*,*e*][1,4]diazepin-11-one and 4-[(2*E*,6*E*)-3,7,11-trimethyl-2,6,10-dodecatrienyl]-naphthalene-1,6-diol.

## Supporting Information

Figure S1 **Proposed MS fragmentation of the product from dibenzodiazepinone and FPP.**
(PDF)

Figure S2 **NMR correlations of the farnesylated dibenzodiazepinone.**
(PDF)

Figure S3 **Proposed MS fragmentation of the product from 1,6-DHN and FPP.**
(PDF)

Figure S4 **NMR correlations of the farnesylated 1,6-DHN.**
(PDF)

Figure S5 **Kinetic data for DzmP and Ssrg_00986.**
(PDF)

Figure S6 **HPLC-MS analysis of incubation of aromatic substrates with FPP and DzmP.**
(PDF)

Figure S7 **Superposition of CloQ and a model of DzmP.**
(PDF)

Table S1 **^1^H NMR and ^13^C NMR data of 1.**
(PDF)

Table S2 **^1^H NMR and ^13^C NMR data of 2.**
(PDF)
