# Supplementary material for: Unusual N-Prenylation in Diazepinomicin Biosynthesis: The Farnesylation of a Benzodiazepine Substrate Is Catalyzed by a New Member of the ABBA Prenyltransferase Superfamily
Source: PLoS One. 2013 Dec 23;8(12):e85707. doi: 10.1371/journal.pone.0085707 (PMC3871700; doi:10.1371/journal.pone.0085707)
Supplement: Figure S1 — Proposed MS fragmentation of the product from dibenzodiazepinone and FPP. (PDF) [file pone.0085707.s001.pdf]

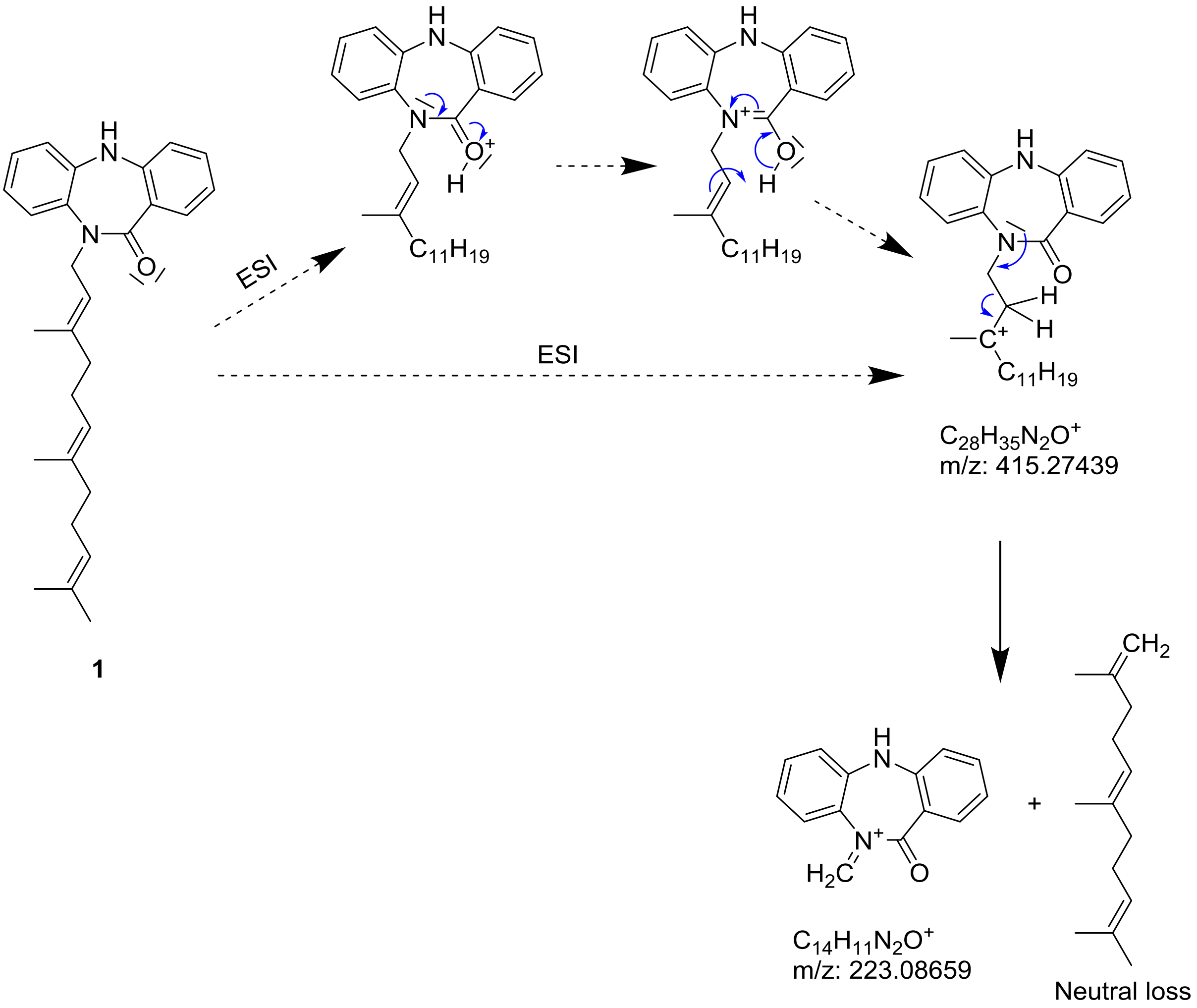

Figure S1. Potential protonation and proposed MS fragmentation mechanism of the product formed under catalysis of DzmP from dibenzodiazepinone and FPP, 5,10-dihydro-10-[(2*E*,6*E*)-3,7,11-trimethyl-2,6,10-dodecatrienyl]-11*H*-dibenzo[*b*,*e*][1,4]diazepin-11-one (**1**).
