# Supplementary material for: Unusual N-Prenylation in Diazepinomicin Biosynthesis: The Farnesylation of a Benzodiazepine Substrate Is Catalyzed by a New Member of the ABBA Prenyltransferase Superfamily
Source: PLoS One. 2013 Dec 23;8(12):e85707. doi: 10.1371/journal.pone.0085707 (PMC3871700; doi:10.1371/journal.pone.0085707)
Supplement: Figure S2 — NMR correlations of the farnesylated dibenzodiazepinone. (PDF) [file pone.0085707.s002.pdf]

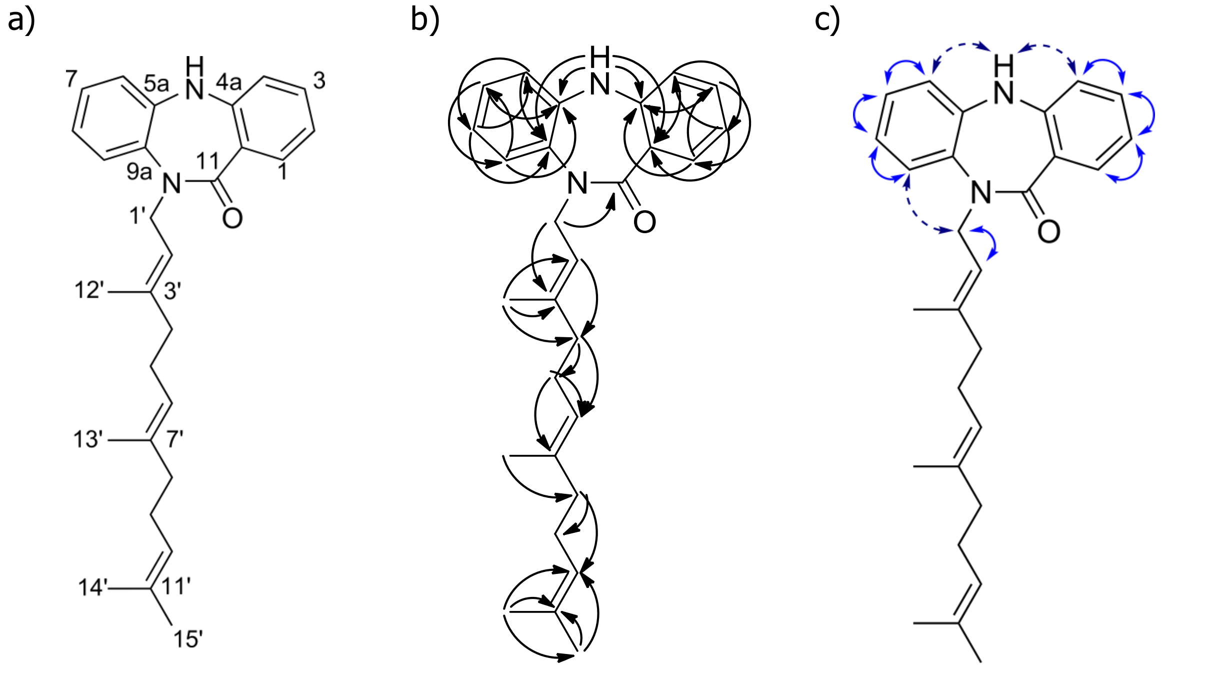


Figure S2. NMR correlations of the farnesylated dibenzodiazepinone **1**. a) Numbering of atoms; b) selected $^{1}H$ HMBC correlations and c) selected $^{1}H$-$^{1}H$ COSY (solid arrows) and NOESY (dashed arrows) correlations.
