# Supplementary material for: Unusual N-Prenylation in Diazepinomicin Biosynthesis: The Farnesylation of a Benzodiazepine Substrate Is Catalyzed by a New Member of the ABBA Prenyltransferase Superfamily
Source: PLoS One. 2013 Dec 23;8(12):e85707. doi: 10.1371/journal.pone.0085707 (PMC3871700; doi:10.1371/journal.pone.0085707)
Supplement: Figure S3 — Proposed MS fragmentation of the product from 1,6-DHN and FPP. (PDF) [file pone.0085707.s003.pdf]

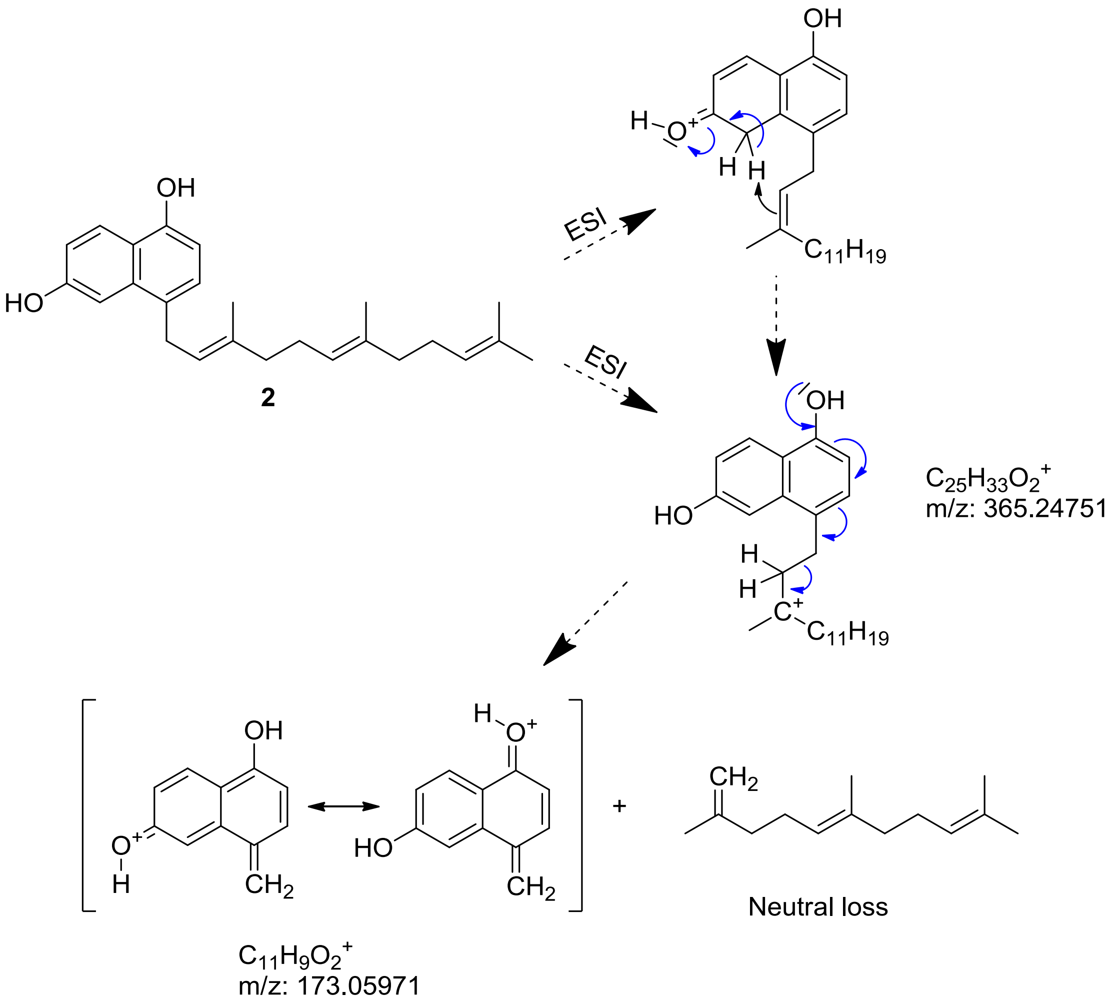

Figure S3. Potential protonation and proposed MS fragmentation mechanism of the product formed under catalysis of DzmP from 1,6-DHN and FPP, 4-[(2*E*,6*E*)-3,7,11-trimethyl-2,6,10-dodecatrienyl]-naphthalene-1,6-diol (**2**).
