# Supplementary material for: Unusual N-Prenylation in Diazepinomicin Biosynthesis: The Farnesylation of a Benzodiazepine Substrate Is Catalyzed by a New Member of the ABBA Prenyltransferase Superfamily
Source: PLoS One. 2013 Dec 23;8(12):e85707. doi: 10.1371/journal.pone.0085707 (PMC3871700; doi:10.1371/journal.pone.0085707)
Supplement: Figure S4 — NMR correlations of the farnesylated 1,6-DHN. (PDF) [file pone.0085707.s004.pdf]

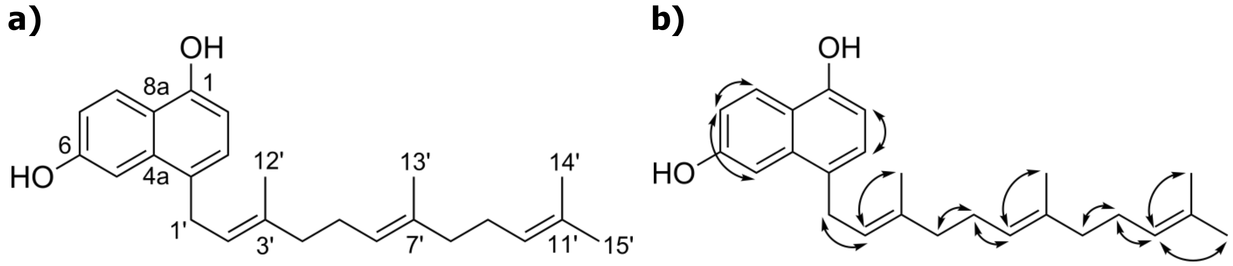

Figure S4. NMR correlations of the farnesylated 1,6-DHN **2**. a) Numbering of atoms and b) selected ^{1}H-^{1}H COSY correlations.
