# Supplementary material for: Unusual N-Prenylation in Diazepinomicin Biosynthesis: The Farnesylation of a Benzodiazepine Substrate Is Catalyzed by a New Member of the ABBA Prenyltransferase Superfamily
Source: PLoS One. 2013 Dec 23;8(12):e85707. doi: 10.1371/journal.pone.0085707 (PMC3871700; doi:10.1371/journal.pone.0085707)
Supplement: Figure S5 — Kinetic data for DzmP and Ssrg_00986. (PDF) [file pone.0085707.s005.pdf]

	DzmP	Ssrg_00986
FPP constant, 1,6-DHN variable	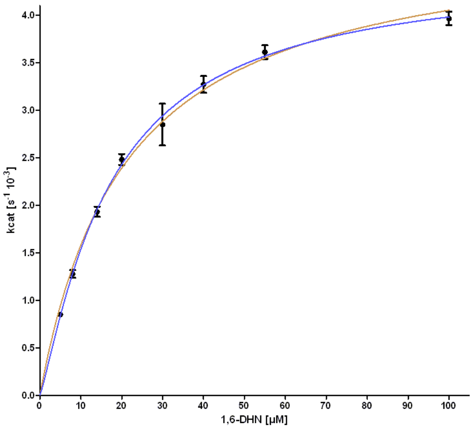	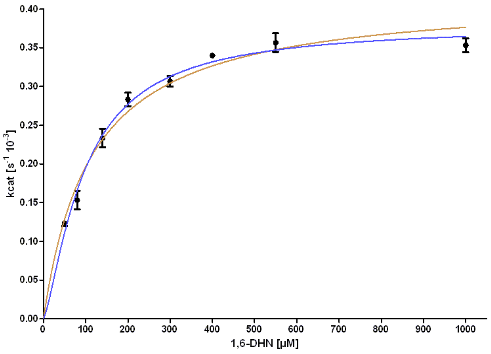
FPP constant, dibenzodi-azepinone variable	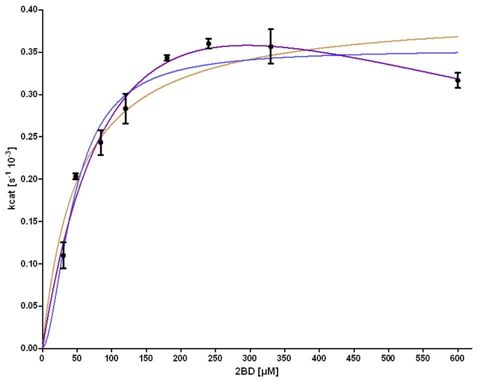	
1,6-DHN constant, FPP variable	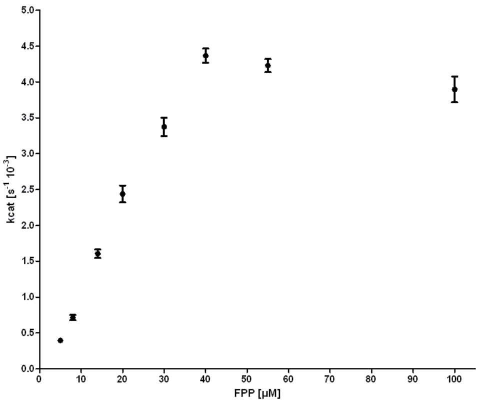	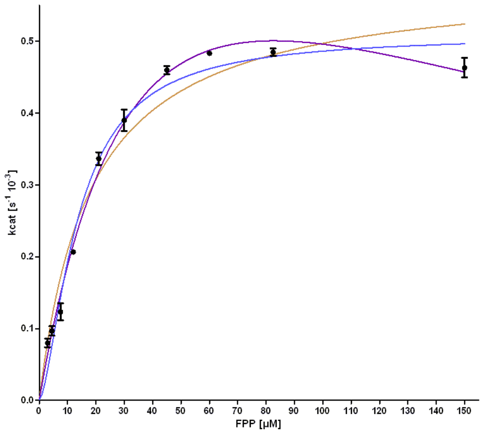
1,6-DHN constant, GPP variable	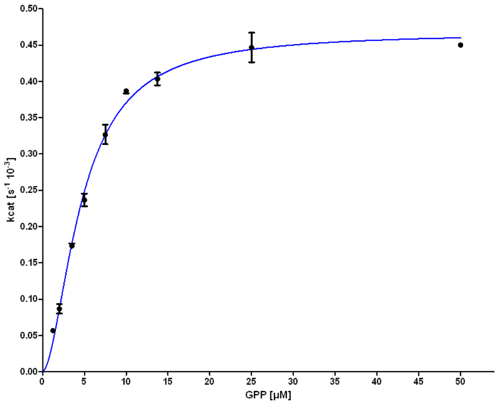	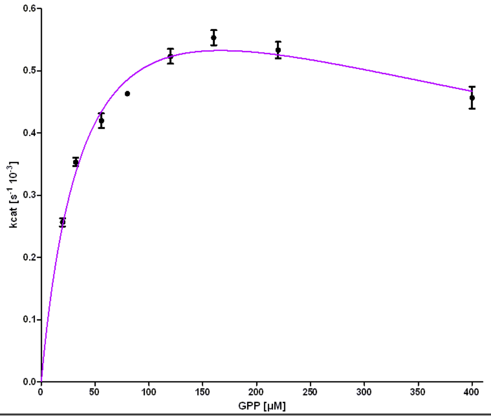

Figure S5. Kinetic data for DzmP and Ssrg_00986. Brown line, Michaelis-Menten equation; blue line, sigmoidal equation; purple line, substrate inhibition equation.
