# Supplementary material for: Unusual N-Prenylation in Diazepinomicin Biosynthesis: The Farnesylation of a Benzodiazepine Substrate Is Catalyzed by a New Member of the ABBA Prenyltransferase Superfamily
Source: PLoS One. 2013 Dec 23;8(12):e85707. doi: 10.1371/journal.pone.0085707 (PMC3871700; doi:10.1371/journal.pone.0085707)
Supplement: Figure S6 — HPLC-MS analysis of incubation of aromatic substrates with FPP and DzmP. (PDF) [file pone.0085707.s006.pdf]

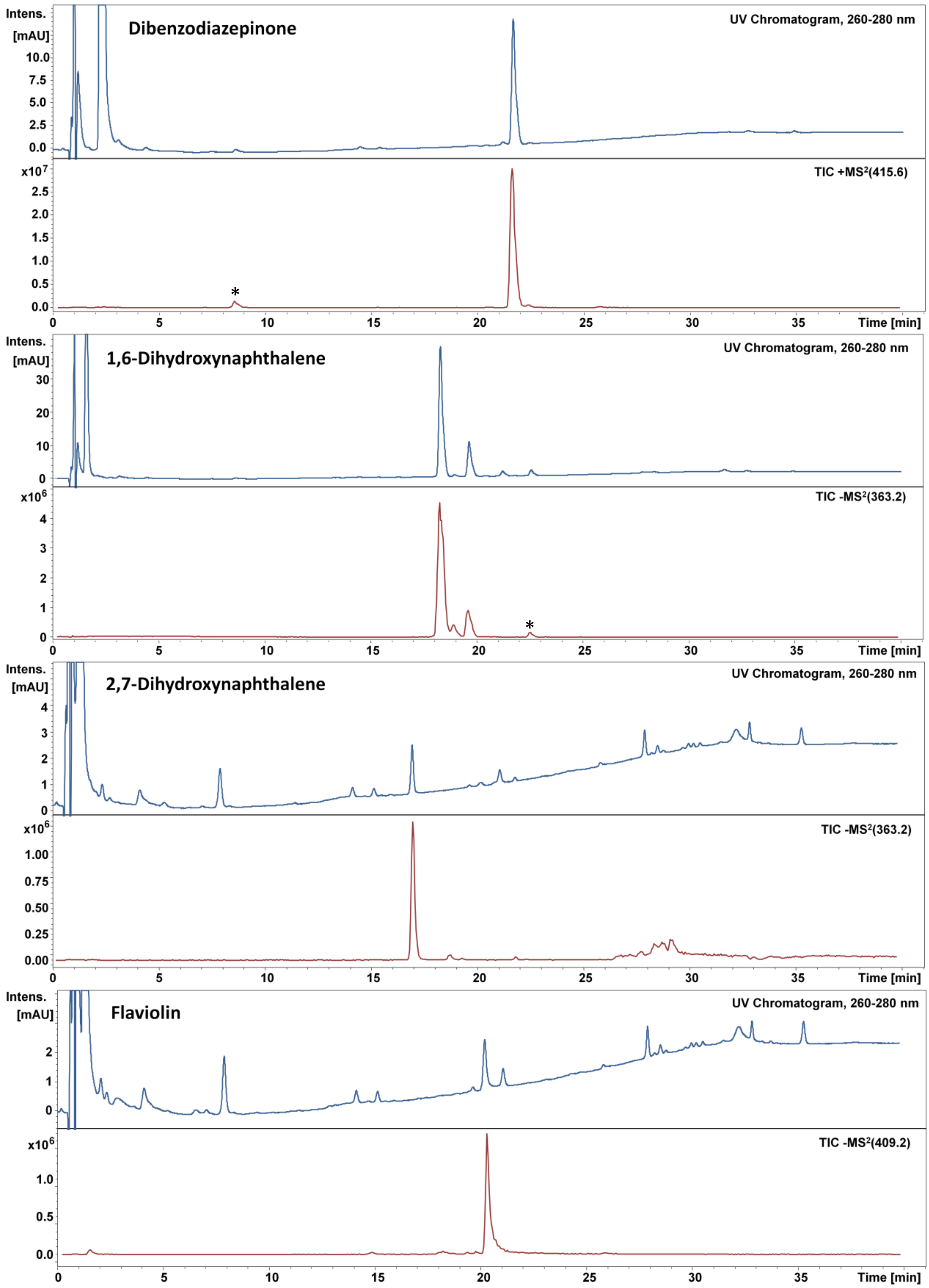

Intens. [mAU]
Dibenzodiazepinone
UV Chromatogram, 260-280 nm
10.0
7.5
5.0
2.5
0.0
x10^7
TIC +MS²(415.6)
2.5
2.0
1.5
1.0
0.5
0.0
*
0
5
10
15
20
25
30
35
Time [min]
Intens. [mAU]
1,6-Dihydroxynaphthalene
UV Chromatogram, 260-280 nm
30
20
10
0
x10^6
TIC -MS²(363.2)
4
3
2
1
0
*
Time [min]
Intens. [mAU]
2,7-Dihydroxynaphthalene
UV Chromatogram, 260-280 nm
4
3
2
1
0
x10^6
TIC -MS²(363.2)
1.00
0.75
0.50
0.25
0.00
Time [min]
Intens. [mAU]
Flaviolin
UV Chromatogram, 260-280 nm
2
1
0
x10^6
TIC -MS²(409.2)
1.0
0.5
0.0
Time [min]

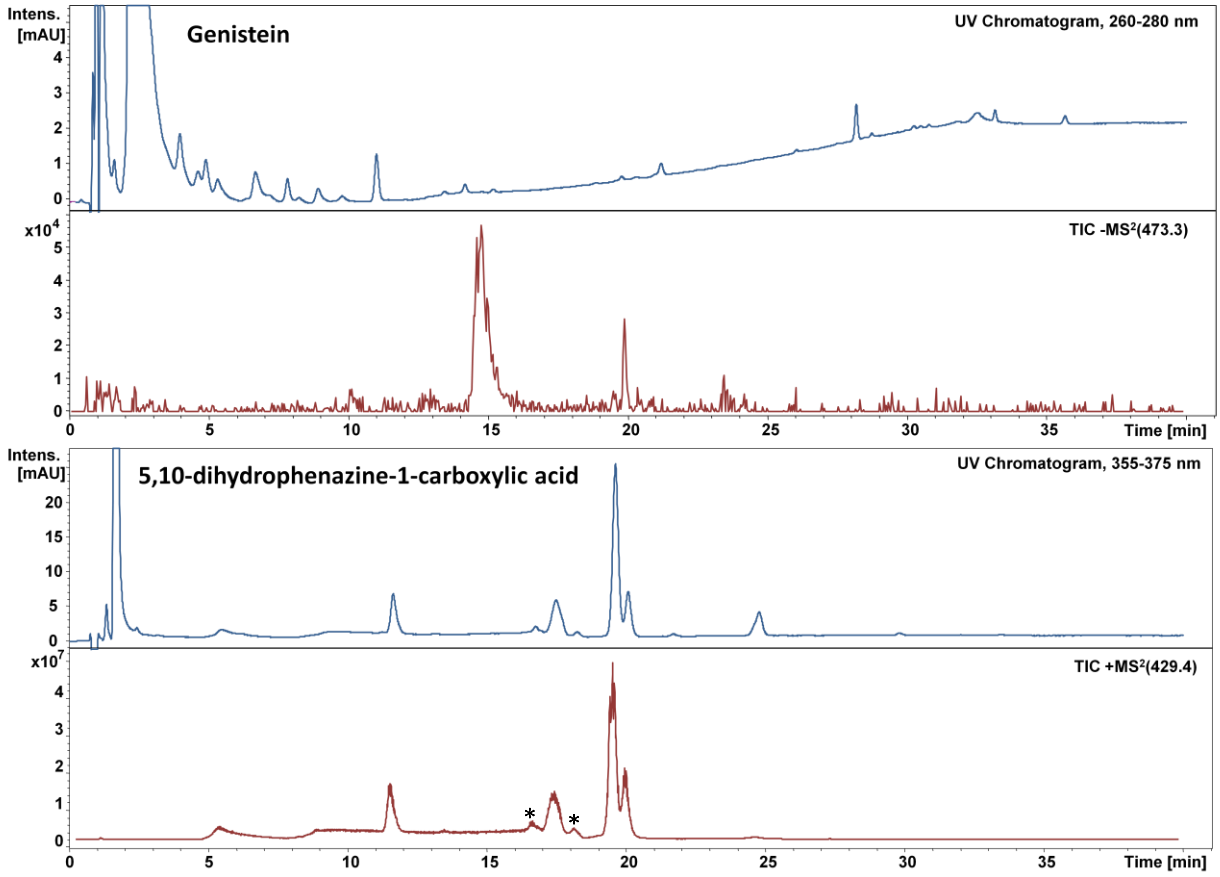


Figure S6. HPLC-MS chromatograms of the incubation of different aromatic substrates with FPP and DzmP. Blue line, HPLC-UV absorbance; brown line, intensity of total ion count (TIC) from MS-MS experiment of the respective mass of the proposed product; asterisks, peaks showing the mass as the proposed product, but not the same fragmentation pattern.
