# Supplementary material for: Unusual N-Prenylation in Diazepinomicin Biosynthesis: The Farnesylation of a Benzodiazepine Substrate Is Catalyzed by a New Member of the ABBA Prenyltransferase Superfamily
Source: PLoS One. 2013 Dec 23;8(12):e85707. doi: 10.1371/journal.pone.0085707 (PMC3871700; doi:10.1371/journal.pone.0085707)
Supplement: Figure S7 — Superposition of CloQ and a model of DzmP. (PDF) [file pone.0085707.s007.pdf]

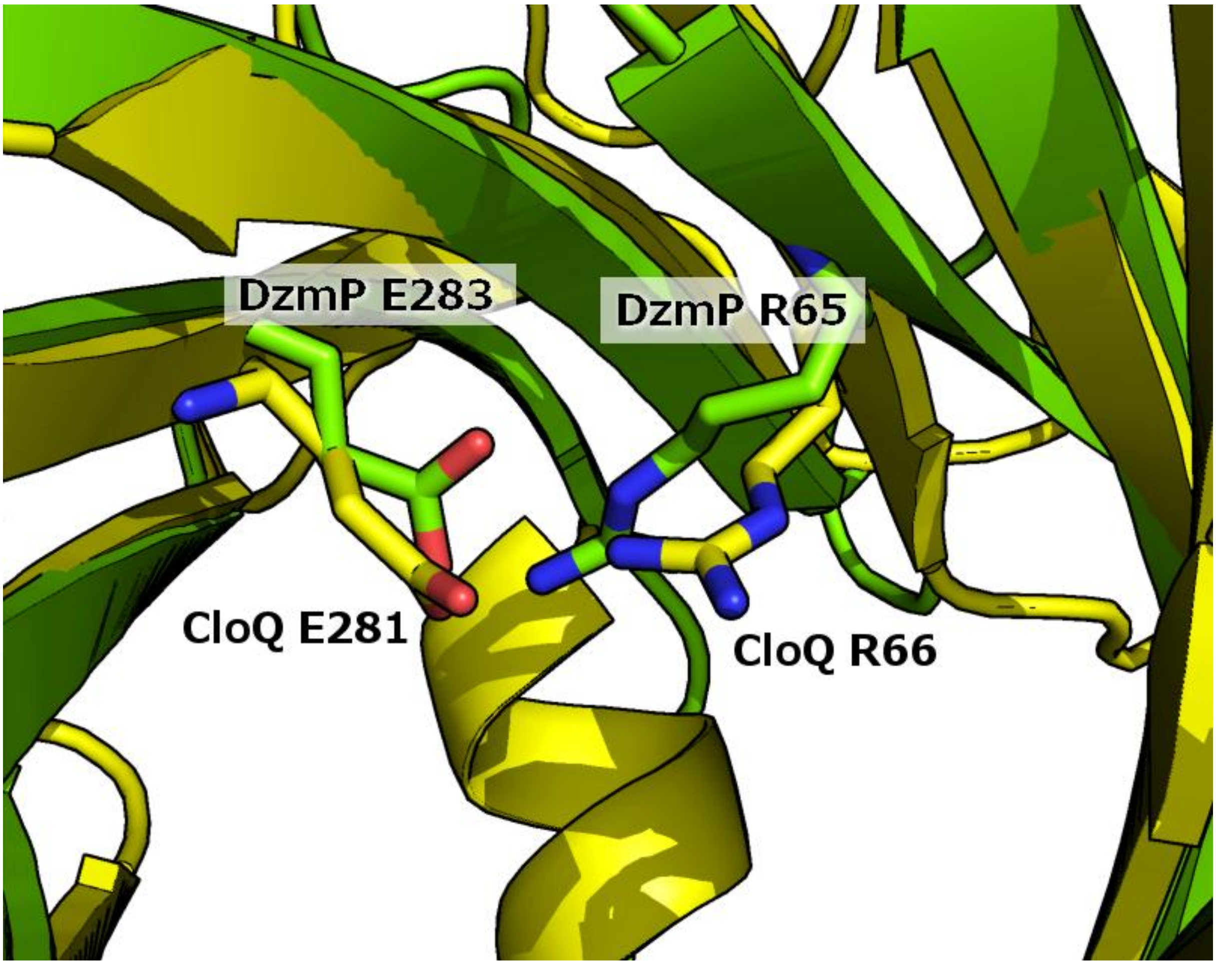

Figure S7. Superposition of CloQ (yellow, PDB: 2XLQ) and a model of DzmP (green). Residues which have previously been suggested to limit the chain length of the isoprenoid substrate of CloQ to five carbons (DMAPP) are shown. However, the same residues are now found to be conserved in DzmP which transfers FPP (15 carbons). Atoms are colored red for oxygen and blue for nitrogen.
