# Supplementary material for: Unusual N-Prenylation in Diazepinomicin Biosynthesis: The Farnesylation of a Benzodiazepine Substrate Is Catalyzed by a New Member of the ABBA Prenyltransferase Superfamily
Source: PLoS One. 2013 Dec 23;8(12):e85707. doi: 10.1371/journal.pone.0085707 (PMC3871700; doi:10.1371/journal.pone.0085707)
Supplement: Table S1 — 1H NMR and 13C NMR data of 1. (PDF) [file pone.0085707.s008.pdf]

Table S1. ^{1}H NMR (600.1 MHz) and ^{13}C NMR (150.9 MHz) data of **1** measured in d6-DMSO.

Position	^{1}H NMR data<br>δ_H [ppm], integral, multiplicity, *J* [Hz]	^{13}C NMR data<br>δ_C [ppm]
1	7.60, 1H, dd, *J* = 7.7, 1.6	132.0
2	6.93, 1H, ddd, *J* = 7.7, 7.5, 1.1	121.4
3	7.31, 1H, ddd, *J* = 8.6, 7.5, 1.6	132.3
4	7.04, 1H, m	118.7
4a	-	152.0
5	7.82, 1H, bs	-
5a	-	145.0
6	7.10, 1H, dd, *J* = 7.9, 1.6	120.4
7	7.04, 1H, m	125.7
8	7.00, 1H, ddd, *J* = 7.9, 7.6, 1.6	123.3
9	7.26, 1H, dd, *J* = 7.9, 1.5	123.4
9a	-	134.0
11	-	167.5
11a	-	124.6
1‘	4.48, 2H, d, *J* = 6.3	47.8
2‘	5.22, 1H, tq, *J* = 6.1, 1.1	121.4
3‘	-	137.4
4‘	1.94, 2H, m	38.9
5‘	1.97, 4H, m (with 9′)	25.8
6‘	5.01, 2H, m (with 10′)	124.1
7‘	-	134.5
8‘	1.88, 2H, t, *J* = 7.0	39.2
9‘	1.97, 4H, m (with 5′)	26.2
10‘	5.01, 2H, m (with 6′)	123.6
11‘	-	130.6
12‘	1.63, 3H, s	16.1
13‘	1.52, 3H, s	15.8
14‘	1.49, 3H, s	17.5
15‘	1.59, 3H, s	25.5
