# Supplementary material for: Unusual N-Prenylation in Diazepinomicin Biosynthesis: The Farnesylation of a Benzodiazepine Substrate Is Catalyzed by a New Member of the ABBA Prenyltransferase Superfamily
Source: PLoS One. 2013 Dec 23;8(12):e85707. doi: 10.1371/journal.pone.0085707 (PMC3871700; doi:10.1371/journal.pone.0085707)
Supplement: Table S2 — 1H NMR and 13C NMR data of 2. (PDF) [file pone.0085707.s009.pdf]

Table S2. ^{1}H NMR (600.1 MHz) and ^{13}C NMR (150.9 MHz) data of **2** measured in d3-acetonitrile.

Position	^{1}H NMR data<br>δ_H [ppm], integral, multiplicity, *J* [Hz]	^{13}C NMR data<br>δ_C [ppm]
1	-	152.3
1-OH	7.22, 2H with 6-OH, bs	-
2	6.58, 1H, d, *J* = 7.6	106.2
3	7.07, 1H, d, *J* = 7.6	127.2
4	-	128.3
4a	-	135.6
5	7.20, 1H, d, *J* = 2.4	107.0
6	-	156.1
6-OH	7.22, 2H with 1-OH, bs	
7	7.03, 1H, dd, *J* = 9.0, 2.4	117.2
8	8.05, 1H, d, *J* = 9.0	125.1
8a	-	120.8
1‵	3.56, 2H, d, *J* = 7.0	31.9
2‵	5.32, 1H, tq, *J* = 7.0, 1.1	124.5
3‵	-	136.6
4‵	2.06, 2H, m	40.3
5‵	2.10, 2H, m	27.1
6‵	5.11, 1H, tq, *J* = 7.0, 1.1	125.3
7‵	-	135.9
8‵	1.91, 2H, m	40.3
9‵	1.99, 2H, m	27.4
10‵	5.06, 1H, m	125.3
11‵	-	132.1
12‵	1.79, 3H, s	16.3
13‵	1.56, 3H, s	16.1
14‵	1.55, 3H, s	17.7
15‵	1.63, 3H, s	25.8
